# Annotations

**Published:** 1906-02-03

**Authors:** 


### ANNOTATIONS.

### Evening Attendance at Dispensaries.

The question of evening attendance at dispensaries has often arisen, the last occasion being at Exeter. The Exeter Dispensary has been established for nearly ninety years, and provides medical attendance and medicine to persons in receipt of 20s. weekly and under, their wives, and children. No persons receiving parochial relief, or belonging to a club, or entitled to gratuitous medical relief elsewhere, are, however, admissible. It appears to be the practice for the Weekly Committee, on the recommendation of a Governor, to admit other cases at their discretion. The question of evening attendance largely depends upon the class of patients received at a dispensary. At Exeter, if the rules are strictly adhered to, the number seeking relief must be severely restricted, for the patients should be of the poorest. The more strictly the rules are enforced, the more reason may there be for meeting the circumstances of the poorest and most deserving patients by affording them an opportunity to be examined and prescribed for in the evening, so as to enable them to husband their slender resources as much as possible. As a general rule, however, there can be no doubt that it is undesirable for free dispensaries to provide evening attendance and medicine to patients, when, as at Exeter, it is the rule to visit patients at their own homes when they are too ill to attend at the dispensary. The reason is, that if a dispensary is made in all respects to fulfil the conditions which obtain, in ordinary practice, the inducements offered for abuses are greatly enhanced. By such a step the free dispensaries might in time become the worst-abused of medical charities. At a provident dispensary the conditions are different, because there the patients do pay for the relief they require, and they are entitled to have such arrangements made as will best suit their convenience. It is the usual practice for a provident dispensary to provide for evening attendances. The question is one which should be determined on the merits and circumstances of the patients at each centre served by a dispensary. There need be no difficulty, providing the rules are clear and adequate, when they are invariably and strictly enforced.

### The Parish Doctor and His Pay.

An application made by the medical officer of the Bromsgrove Workhouse for an increase of salary brought forward some interesting points in the payment of those medical men who work under the Guardians of the poor. The medical officer in question receives £40 a year for his services, out of which he has to provide medicines; and some of the Guardians thought that he needed an increase. One Guardian pointed out that the Chaplain received an equal salary for his work, and out of this had to provide nothing which left him poorer in pocket. But this comment was regarded by the majority of those present as a mere flippant personality, and finally the proposal to increase the doctor's salary from £40 to £60 was negatived by nine votes to four. Shortly before this the Guardians of the Clutton Union had received an application from some of their district medical officers that the Guardians should pay for certain expensive medicines which they had sometimes occasion to prescribe; but their clerk reported that it was not the custom of unions to do so, and the application was refused. As a matter of fact, the custom of including the provision of medicines in a doctor's salary is one that is bound to prove unsatisfactory. If the parish doctor is to pay for drugs out of his own pocket, the profit on a laborious and ill-appreciated appointment will fall to zero. If, on the other hand, he is not prepared to face this sacrifice, then diphtheria or some equally deadly disease may work its will without the checks that modern science puts in its way. Very rarely indeed, we believe, does anyone die because the doctor grudged the necessary medicine, but is it fair to ask a doctor, who has as much right to live as a pauper, or even as a Guardian, to provide costly medicines out of his own pocket? Is not the simple solution to pay the doctor for his professional advice, and pay the chemist who supplies the medicines he prescribes at a reasonable rate for his drugs?

### The Motor Madman.

The new House of Commons cannot be expected to legislate in reference to motor-car regulations until the Commission, of which Lord Selby is chairman, has issued its report. But both in the interest of the individual and of the community it is extremely important that any attempts on this side of the Atlantic to emulate the performances which have just taken place in the United States should be strongly condemned. In one case the American owner of a steam-propelled automobile covered a mile in 28 1-5th seconds, and in the other an English amateur, driving a 90-horse-power Napier, covered a hundred miles in one hour fifteen minutes. Both these feats were accomplished in Ormond Beach, Florida; and it is stated that "nobody expected either of the record-breakers to come out of the race alive." One found that his eyes were cut and his face hurt by the rush of wind; and the other, who ran seventy-five miles at top speed without the tyre of one of his rear wheels, was admittedly saved from destruction only by "magnificent steering and presence of mind." It is, we fear, useless to appeal to the motor madman in America, but it may not yet be too late to warn those who are ambitious to pose as his rival here that driving at an excessive rate of speed is perilous to the motorist, even though he may achieve dare-devil deeds and enjoy hair-breadth escapes. Such a pastime cannot be indulged in without serious risks of encroaching upon the integrity of the central nervous system. We think that if this could be more clearly realised there would be less disposition than there is at present to abuse an invention whose use, under sensible conditions, not only affords pleasure and economises time, but may materially contribute to the promotion of health.

